# Editorial: Exploring anesthetic risk: challenges and solutions in veterinary medicine

**DOI:** 10.3389/fvets.2025.1587332

**Published:** 2025-03-17

**Authors:** Karine Portier, Mark Senior

**Affiliations:** ^1^VetAgro Sup, CREFAC, Université de Lyon, Marcy l'Etoile, France; ^2^Centre de Recherche en Neurosciences de Lyon, INSERM, CRNL U1028 UMR5292, Trajectoire, Université Claude Bernard Lyon 1, Bron, France; ^3^School of Veterinary Science, University of Liverpool,Leahurst Campus, Neston, United Kingdom

**Keywords:** anesthesia, anesthetic risks, veterinary medicine, drug interactions, analgesic

The field of veterinary anesthesia is continuing to evolve: however, anesthetic risk remains a critical concern for veterinarians and pet owners alike. As the need for surgical procedures continues to grow, so does the necessity to refine anesthetic protocols, minimize complications, and improve patient safety. No surgical anesthetic intervention is completely benign, and so to improve patient safety we need to evaluate the potential benefit of an intervention against its risk. Understanding anesthetic risk, in simple terms, means understanding the possibility of something bad happening in relation to anesthesia, and that something bad normally means a morbidity or a fatality. Assessing anesthetic risk involves multiple factors, such as species-specific responses, drug interactions, and pre-existing conditions. The studies presented in this Research Topic explore anesthetic risk through a breadth of topics that include providing information on the efficacy and safety of drugs in anesthetized exotic species, uncommon complications in commonly anesthetized species using commonly administered drugs, structured risk assessment methods, and novel strategies to mitigate adverse effects.

One of the significant challenges in veterinary anesthesia is the assessment of pain and analgesic efficacy across diverse species. The study on hydromorphone administration in American alligators (Henke et al.) sheds light on pain management in reptiles, a field where research remains limited. By demonstrating the efficacy of hydromorphone with minimal adverse effects, this research expands our knowledge of species-specific analgesia and highlights the importance of tailored pain management strategies in non-mammalian patients.

In wildlife and conservation medicine, anesthesia is essential for capture and for both medical and research purposes. A study on snow leopard immobilization with ketamine-xylazine (Talukdar et al.) provides valuable insight into the physiological responses of free-ranging animals under emergency conditions. The documented safety and efficacy of this anesthetic combination reinforce its viability in field settings, offering a benchmark for future anesthetic protocols in large wild felids.

Anesthetic-induced complications remain a significant concern, as evidenced by a case report on hyperkalemia in a domestic cat under general anesthesia (Irizarry and Graidlla). The suspected link to propofol infusion syndrome (PRIS) highlights the importance of vigilance in recognizing and managing intraoperative metabolic disturbances. This case serves as a reminder of the unpredictable nature of anesthetic reactions and the need for continuous monitoring and preparedness for rapid intervention.

Risk assessment tools play a vital role in pre-anesthetic evaluation. The introduction of the CHARIOT scoring system for equine anesthesia (Brumund et al.) provides a structured approach to predicting peri-anesthetic morbidity and mortality. While the study included in this Research Topic indicates that CHARIOT has moderate predictive accuracy, refining and validating risk assessment tools remains an ongoing priority to improve anesthetic safety in horses.

Pharmacologic formulations significantly impact anesthetic safety, as illustrated by a study on etomidate in propylene glycol in minipigs (Petrucci et al.). The observed adverse effects, such as hemolysis and laryngeal edema, raise concerns about the suitability of the formulation. This study underscores the necessity of evaluating drug formulations across different species to mitigate unforeseen complications.

Long-term analgesic management also warrants careful consideration. A study on enflicoxib, a COX-2 selective NSAID, explores its efficacy and safety in managing osteoarthritis in dogs (Homedes et al.). The sustained analgesic benefits and lack of significant adverse effects over an extended period suggest that enflicoxib may offer a viable long-term pain management solution for canine patients with osteoarthritis.

Finally, a comparative study of propofol and alfaxalone on canine electrocardiographic parameters highlights the potential cardiac effects of anesthetic agents (Casoria et al.). The findings suggest that both drugs prolong the QTc interval, raising concerns about their proarrhythmic potential. This research underscores the importance of selecting anesthetic agents based on individual patient risk profiles, particularly in animals with pre-existing cardiac conditions.

Taken together, these studies contribute to a more comprehensive understanding of anesthetic risk across a range of species and clinical scenarios. By addressing key challenges in anesthetic management, from species-specific analgesia to risk assessment tools and pharmacologic considerations, this Research Topic fosters advances in veterinary anesthesia. Moving forward, continued research and interdisciplinary collaboration will be essential to refine anesthetic protocols, minimize risk, and improve patient outcomes in veterinary medicine.

